# Immune control of HIV-1 infection after therapy interruption: immediate versus deferred antiretroviral therapy

**DOI:** 10.1186/1471-2334-9-172

**Published:** 2009-10-19

**Authors:** Paola Paci, Rossella Carello, Massimo Bernaschi, Gianpiero D'Offizi, Filippo Castiglione

**Affiliations:** 1 Institute for Computing Applications "Mauro Picone", National Research Council, Rome, Italy; 2 National Institute for Infectious Diseases "Lazzaro Spallanzani", I.R.C.C.S., Rome, Italy; 3 Research Center, San Pietro Hospital, Fatebenefratelli, AFaR, Rome, Italy

## Abstract

**Background:**

The optimal stage for initiating antiretroviral therapies in HIV-1 bearing patients is still a matter of debate.

**Methods:**

We present computer simulations of HIV-1 infection aimed at identifying the *pro et contra *of *immediate *as compared to *deferred *Highly Active Antiretroviral Therapy (HAART).

**Results:**

Our simulations highlight that a prompt specific CD8^+ ^cytotoxic T lymphocytes response is detected when therapy is delayed. Compared to very early initiation of HAART, in deferred treated patients CD8^+ ^T cells manage to mediate the decline of viremia in a shorter time and, at interruption of therapy, the virus experiences a stronger immune pressure. We also observe, however, that the immunological effects of the therapy fade with time in both therapeutic regimens. Thus, within one year from discontinuation, viral burden recovers to the value at which it would level off in the absence of therapy.

In summary, simulations show that immediate therapy does not prolong the disease-free period and does not confer a survival benefit when compared to treatment started during the chronic infection phase.

**Conclusion:**

Our conclusion is that, since there is no therapy to date that guarantees life-long protection, deferral of therapy should be preferred in order to minimize the risk of adverse effects, the occurrence of drug resistances and the costs of treatment.

## Background

Antiretroviral therapy has significantly modified the approach to treatment of the Human Immunodeficiency Virus (HIV) infection. Positive effects of Higly Active Antiretroviral Therapy (HAART) include suppression of plasma viremia below detection level, rising circulating CD4^+ ^T cell count, reduction of the incidence of acquired immunodeficiency syndrome (AIDS) and of death [1-3]. It is still unclear, however, what the "ideal moment" is for initiating HAART. An apparent advantage of any antiretroviral treatment started during the early phases of HIV-1 infection (*i.e*., immediate or very early therapy) is to preserve immune function and to reduce the risk of viral transmission [1,4-6]. However immunological benefit from HAART has been shown also in asymptomatic patients with chronic infection (*i.e*., deferred therapy) by documented restoration of the naïve CD4^+ ^T cell count and improved antigen-specific immunity [7]. Possible drawbacks to very early initiation of HAART, include prolonged exposure to antiretroviral therapy without known clinical benefit, exposure which could result in drug toxicities and development of antiretroviral drug resistance, the need for continuous therapy with strict adherence and associated adverse effect on quality of life and, last but not least, increased costs [5]. The demand of new therapeutic strategies and the urgent need to shed light on the issue of when is the best time for initiating therapy together with the high cost and long follow-up required by *in vivo *testing, provide solid arguments for the use of computational models as predictive tools.

In the present study we resorted to a computer model to forecast the immune/viral dynamics of *in silico *patients treated with HAART either in the acute phase of primary infection or at a later date during the chronic disease. The computational model employed closely reproduces the hallmarks of HIV-1 infection for both drug-free (details are described elsewhere [8]) and treated patients. We have compared our results with a cohort of twenty-two patients undergoing immediate therapy and used the simulator to asses the benefits of delayed HAART initiation.

Our results suggest that deferral of HAART favors HIV specific cytotoxic T lymphocytes while minimizing toxic side-effects, when compared to very early therapeutic intervention.

## Results

### Subjects analysis

We started from the analysis of the cohort data of naïve patients that received HAART (the therapeutic regimen included protease inhibitors, nucleoside reverse transcriptase inhibitors and non-nucleoside reverse transcriptase inhibitors) within six months from primary infection (*i.e*., including both the inflammatory phase and after seroconversion). Table 1 reports clinical information about the cohort data. In Table 2 patients have been classified according to the staging method proposed by Fiebig *et al *[9].

**Table 1:** Subjects with an immediate treatment of acute HIV-1 infection.

	ID	Age	Therapy†	Elapsed days‡	Days on therapy	At diagnosis*			At first interruption		
						CD4	CD8	vRNA × 10^4^	CD4	CD8	vRNA
	Pt 03	19	D4T, 3TC, IDV	14	1775	653	1659	75	809	459	5794
	Pt 05	40	AZT, 3TC, IDV	61	1657	444	633	100	867	476	*<*50
	Pt 29	35	AZT, 3TC, NFV	14	1069	1103	1964	31	741	844	*<*50
	Pt 33	19	AZT, 3TC, IDV	66	925	424	991	0.13	458	474	147
Arm A	Pt 35	39	AZT, 3TC, EFV	1	1041	522	1205	78	919	607	496
	Pt 37	52	AZT, 3TC, EFV	0	1371	545	1233	3.9	901	384	*<*50
	Pt 58	26	AZT, 3TC, EFV	3	442	768	1632	50	1451	1580	*<*50

	Pt 06	24	AZT, 3TC, IDV	32	1620	882	4146	4.1	754	726	*<*50
	Pt 18	30	AZT, 3TC, IDV	26	635	1319	1810	15	1718	825	*<*80
	Pt 24	34	AZT, 3TC, IDV	3	1231	507	3162	78	513	1067	*<*50
	Pt 31	57	AZT, 3TC, EFV	19	1102	322	243	490	1154	1165	*<*50
Arm B	Pt 41	35	AZT, 3TC, EFV	12	705	568	278	130	1326	940	*<*50
	Pt 45	27	AZT, 3TC, EFV	5	488	307	1032	130	872	506	*<*50
	Pt 53	27	AZT, 3TC, Lop/rit	16	503	341	779	18.9	545	529	150

	Pt 04	25	AZT, 3TC, IDV	6	1561	603	1288	1.6	1047	1015	88
	Pt 19	41	AZT, 3TC, IDV	13	1480	1338	716	10	1113	447	*<*50
	Pt 28	25	AZT, 3TC, NFV	7	325	281	860	190	863	485	*<*80
	Pt 32	36	AZT, 3TC, EFV	15	1384	409	1448	4.3	960	331	*<*50
	Pt 72	20	AZT, 3TC, LPV	7	318	827	386	19	1005	751	*<*50
	Pt 81	33	AZT, 3TC, NVP	33	745	412	264	32.5	522	461	68
	Pt 85	27	AZT, 3TC, Lop/rit	46	717	326	669	50	855	1436	*<*50
	Pt 92	36	3TC, Lop/rit, TNF	0	455	616	3774	46.3	1027	913	*<*50

Clinical information about the twenty-two patients selected at the Clinical Department of the National Institute for Infectious Disease "L. Spallanzani" in Rome. All subjects received HAART within six months from primary infection.

**†**D4T, staduvine; 3TC, lamivudine; IDV, Indinavir; AZT, Zidovudine; NFV, nelfinavir; EFV, Efavirenz; NVP, nevirapine; TNF, Tenofovir; Lop, lopinavir; Rit, Ritonavir. **‡ **Days elapsed from diagnosis (enrollment) to initiation of HAART. *CD4 and CD8 are per microlitre of plasma, viremia is per millilitre of plasma.

**Table 2:** Stages of early HIV infection.

ID	HIV-RNA	p24 antigenemia	HIV EIA Ab-reactive	Wb	Wb p24	Wb gp41	Wb gp160	Wb p31	Wb p55	Wb p66	Wb p17	Stage
Pt 03	+	+	+	+	+		+	-	+	+		V

Pt 04	+	-	+	+	+	+	+	+		+	+	VI

Pt 06	+	+	+	+	+		+	+	+		+	VI

Pt 18	+	-	+	+	+		+	+	+	+	+	VI

Pt 19	+		+	+	+		+	-				V

Pt 24	+	+	-	-								II

Pt 28	+	+	+	ind.	+				+			IV

Pt 29	+	-	+	+	+		+	-	+			V

Pt 31	+	+	+	ind.								IV

Pt 32	+	-	+	+	+		+	-				V

Pt 33	+		+	+	+		+	-	+			V

Pt 35	+		+	ind.	+				+			IV

Pt 37	+	+	+	ind.								IV

Pt 45	+	+	+	+	+		+	-				V

Pt 53	+	+	+	+	+		+	+	+	+	+	VI

Pt 58	+	+	+	+	+		+	-	+		+	V

Pt 72	+		+	?				-	+	+		V

Pt 81	+		+	+	+	+	+	-	+	+	+	V

Pt 85	+	+	+	-								III

Patients in Table 1 classified according to the staging method in [9].

Table 2: Because data is missing, Pt 5, Pt 41 and Pt 92 are not reported in this table. Wb means Western blot pattern. Indeterminate means the presence of HIV-1 specific Wb bands that fail interpretative criteria for reactive Wb defined as reactivity to two of the following three bands: p24, gp41, gp120/160. Positive means reactive Western blot to two of the three bands: p24, gp41, gp120/160.

**Figure 1:**
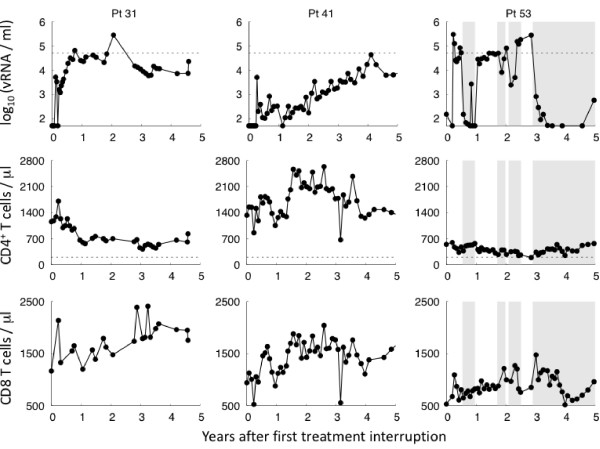
**Time evolution of the virological parameters for STI subjects**. Plasma viremia, CD4^+ ^T cell and CD8 T cell response are plotted for three subjects of the cohort data. They were started on long courses of HAART within the first six months of primary infection (from left to right 3, 2 and 1.5 years, respectively). Then, they were enrolled in a structured interruption protocol (arm B). The therapy resumption was required for a viral load ≥ 50,000 copies/*ml *(dashed line). The shaded area indicates the reinstitution of therapy. They managed to control viremia after the initial treatment interruption for at least one month.

**Figure 2:**
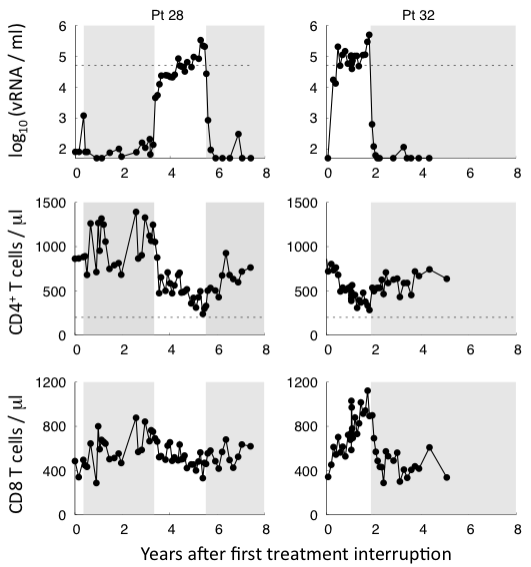
**Time evolution of the virological parameters for unplanned therapy interruption subjects**. Plasma viremia, CD4^+ ^T and CD8 T cell response are plotted for two subjects of the cohort data. They were started on long courses of HAART during the acute phase of the primary infection (from left to right 1 and 3.8 years, respectively). They then underwent unplanned therapy interruptions, with resumption based on medical consensus. The shaded area indicates the reinstitution of therapy. They controlled viremia after the initial treatment interruption for at least one month.

After a variable period of therapy (average: 3 ± 1 years), all subjects were enrolled in Structured Therapy Interruption (STI). For the majority of the subjects, plasma viremia reverts to detectable levels in a median time of ten days following the initial HAART interruption. Despite this rapid viral rebound, five subjects managed spontaneously to control viremia maintaining their plasma virus load below the detection limit for a longer time (see Figure 1 and 2). However, after 1 month, the virus was again detectable and the patients were enrolled in STI protocols. The CD4^+ ^T cell response declined in absence of treatment though remaining well above the level of 200 cells/*μl *defined as the onset of full-blown disease. However, with re-institution of HAART the CD4^+ ^T cell response rose again. Interestingly, in all five subjects exposure to the virus is associated with a substantial increase of the cytotoxic response.

### In silico study of early versus deferred HAART

**Figure 3:**
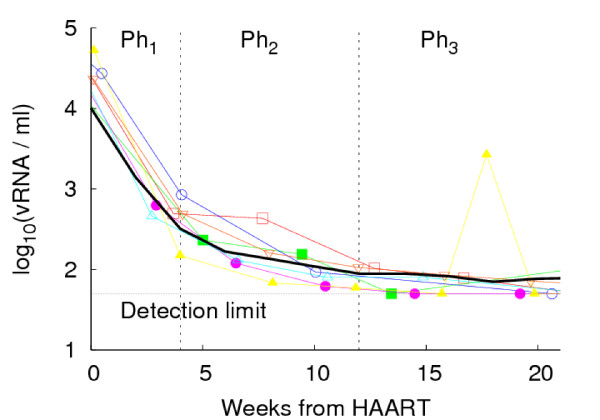
**Pharmacodynamics of HIV-1**. The effect of the antiretroviral treatment is to suppress viremia below the level of detection. This occurs in the first weeks after start of therapy. In particular, within two/three weeks there is an exponential decline of viremia of about two orders of magnitude. This rapid ablation of initial viremia reflects the decay of productively infected cells due to the action of reverse transcripatase inhibitors. The decline then gets slower and slower. This is mainly due to the action of the protease inhibitors on infected cells. Finally, the viral decline levels off, as a result of sub-optimal drug effectiveness, reservoirs of virus-producing cells that are unaffected by the drug, or the emergence of resistant virus strains. Solid line represents the average behavior of hundreds *in silico *patients, whereas colored dots correspond to selected *in vivo *patients.

We used the cohort data, in particular the time window before patients were enrolled in the STI protocol, to set the initial conditions of the model. First of all, we performed computer simulations in which we started a long course of HAART (2.5 years) within six months from primary infection (*i.e*., including both the inflammatory phase and after seroconversion). Then, we fixed immunological parameters at therapy start time on the basis of the average values measured in *in vivo *patients: (5.8 ± 0.2) RNA copies/*ml *(in logarithmic scale), (870 ± 50) CD4 cells/*μl *and (430 ± 50) CD8 cells/*μl*. Finally, the drug control effect of HAART is to suppress viremia, during the first ten weeks, below the detection level. This occurs in three phases with a specific time table [10,11]. We have implemented the model to take into account this dynamics by considering the probability that a virus infects a cell during HAART, follows a characteristic power low decay. The average viremia of hundreds of *silico *patients has been compared with those obtained from *in vivo *patients whose viral load was known in the same time window (see Figure 3).

**Figure 4:**
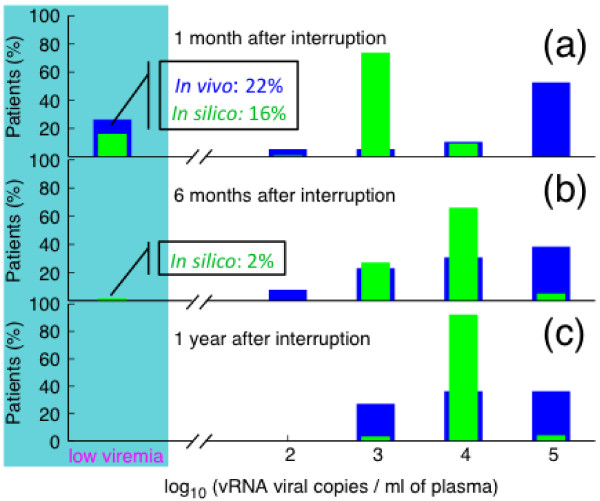
**Viral rebound after stopping immediate HAART**. Percentage of patients with a given level of plasma viremia after stopping HAART as results from *in vivo *studies (blue rectangle) and from simulations (green rectangle). The viremia has been monitored in three different stages after discontinuation. From top to bottom: 1 month, 6 months and 1 year after therapy termination, respectively. Both *in vivo *and *in silico *patients were started on HAART during primary infection. The shadowed area (low viremia *<*50 RNA copies/*ml*) corresponds to the percentage of patients that manage spontaneously to control viremia after the initial interruption of therapy. We predict 16% of successful cases of immune control, which is in good agreement with the 22% obtained by clinical observations.

**Figure 5:**
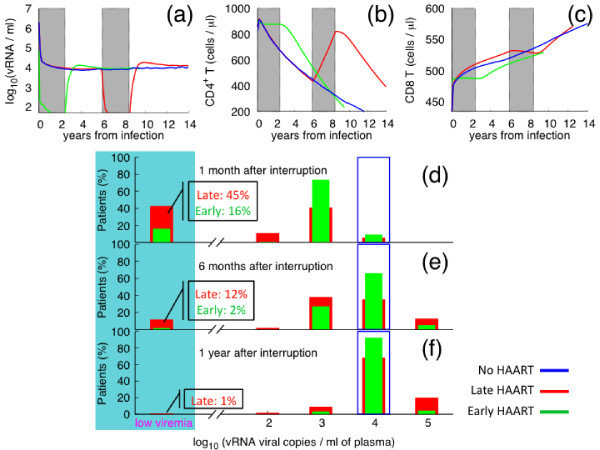
**Viral rebound after stopping deferred HAART**. The drug control effect of HAART on plasma viremia is shown for treated subjects and compared with the viral production in absence of therapy. In the upper panel, from left to right, the viral load, CD4^+ ^and CD8^+ ^T cell count are plotted as function of years from primary HIV-1 infection. In the lower panel, the histogram of the viral rebound after the first interruption of HAART is shown for three different stages (from top to bottom: 1 month, 6 months and 1 year after therapy termination, respectively) and for *in silico *patients receiving either immediate (green histogram) or deferred therapy (red histogram). The shadowed area (low viremia *<*50 RNA copies/*ml*) corresponds to the successful cases of immune control in drug-free period. We find that in delayed treatment 45% of *in silico *patients manage to spontaneously control viremia after the first month of therapy interruption and the virus has yet completely rebound one year after discontinuation, approaching the value at which it would level off in the absence of treatment (blue open rectangle).

Due to the inability to eliminate virus infected cells, antiretroviral treatment fails to eradicate the infection and cessation of suppressive therapy will, most likely, be accompanied by viral rebound. We have analyzed the pace of viral rebound and the ability of early-treated *in silico *patients to spontaneously control HIV-1 replication, in relation to the outcomes of *in vivo *studies. We found that during the first month after treatment interruption, despite viral rebound, 16% of *in silico *patients are able to achieve, at least, a transient steady state off therapy, with viral load below the limit of detection (Figure 4 panel a, green rectangle in shadowed area). Our results are in good agreement with cohort data that give about 22% of successful cases of immune control (panel a, blue rectangle in shadowed area). After that, the virus becomes sparser but still detectable and only 2% of *in silico *patients manage spontaneously to control viremia six months after the initial discontinuation (shadowed area in panel b). Finally, the virus rebounds in one year off therapy, to reach the value at which it would level off in the absence of treatment (green rectangle in panel c). These findings are confirmed by *in vivo *data in which the median viremia one year after stopping HAART is about 10,000 RNA copies/*ml *(*i.e*., log(RNA/*ml*) = 4, blue rectangles in panel c). Despite considerable advances in the understanding of the interplay between HIV-1 and its host, the effect of antiretroviral treatment on the developing immune response remains elusive. During the acute stage of HIV-1 infection in untreated subjects, viral load expands exponentially and antiviral immune defenses are still developing [12]. Once the HIV-specific immune response has been established, viral load usually decreases until a viral set point is reached. It has been shown that initiation of HAART before the attainment of the natural equilibrium of virus load may blunt the natural anti-HIV immune activity [12]. We have investigated this issue and compared the results with those obtained *in silico *when HAART is delayed. In particular, in order to determine the optimal time to initiate HAART, we performed simulations in which we started a long course of HAART (2.5 years) either during seroconversion (Figure 5, green lines in panels a, b and c) or during the chronic stage of the disease (red lines of the same panels). In deferred treatment, we decided to start therapy when viral load was (4.0 ± 0.2) copies/*ml *(in logarithmic scale) and the CD4^+ ^T cell count was (400 ± 50) cells/*μl*. Moreover, we compared CD4^+ ^T cell count and viremia between treated and untreated *in silico *patients (blue lines in panels a, b and c).

Although the impact of a therapeutic intervention on plasma viremia is well documented [13-15], much less is known about the rapidity of decline with respect to timing of therapy. Simulations reveal that with late treatment plasma viremia declines more rapidly, approaching the limit of detection within the first ten weeks, whereas viral load reaches the undetectable level only after six months when therapy is started at an early stage of disease (see Figure 3). Moreover, the virus rises more slowly in deferred treated patients. After one year, a small percentage (1%) of *in silico *patients still manage to spontaneously control viremia, whereas the virus rebounds completely one year after therapy discontinuation in early treated infected persons (Figure 5, shadowed area in panel f). The decresing number of *in silico *patients that spontaneously control viremia, correlates in time strictly with the restoration of the viral set point reached within one year of the termination of HAART. By that time, the efficacy of antiretroviral drugs on viremia is almost vanished and the virus reverts to the value at which it would level off in the absence of treatment (blue rectangle in panel d, e and f).

The evolution of the immune response and, in particular, the kinetics of CD4^+ ^and CD8^+ ^T cells in patients treated with HAART, are subject of heated debate [12,16]. Our results confirm the expectation that initiation of HAART during seroconversion leads to the preservation of HIV-specific T helper cells with the consequent maintenance of specific immunity (Figure 5, green line panel b). In addition, we suggest that *in silico *patients following such an antiviral therapy regimen show a modest loss of CD8^+ ^T cell numbers coincident with a decline in viremia (green line in panel c and a). When therapy ends, the viral load rises again within a year, but in *in silico *patients undergoing early treatment the natural immune activity remains impaired. It thus takes several years to restore the natural equilibrium between the host and its uninvited guest. On the contrary, prior to administration of HAART in chronically infected patients, HIV-1 infection is associated with a failure in T cell homeostasis [17], resulting in a gradual decline in CD4^+ ^T cell numbers (red line panel b), whereas the cytotoxic activity is well developed (red line in panel c). Once therapy starts, CD4^+ ^T lymphocyte counts quickly increase, bringing the host almost to immune restoration.

Simulation of delayed therapy produces enhancements of both functional immune response and immune control of infection. Our interpretation is that, during the period prior to initiation of therapy, the virus elicits a cytotoxic response. This, by contrast, is almost absent in early treatment, as the magnitude of the infection is considerably reduced by the action of the antiretroviral drugs.

## Discussion

The timing of when antiretroviral therapy has to be initiated remains a challenging issue. HAART is costly, it is demanding for both patient and health care provider, and it leads, quite frequently, to adverse events. The clinical benefit of treatment must therefore be weighed against the burden imposed by therapy on the patient and its the side-effects. Immediate treatment has been always advocated as it preserves a higher CD4^+ ^count and prevents potentially irreversible damage to the immune system; it reduces the risk of HIV-associated complications [5]; it may limit the viral dissemination, minimizing harmful virus impact on cellular immune response. However, long-term use of HAART is often accompanied by problems of adherence to therapy regimes, primarily in consequence of intolerance to drugs and serious side effects. Furthermore, long-term use of drugs when adherence is poor is classically associated with the development of viral drug resistance because of incomplete viral suppression, resulting in the loss of future treatment options. Hence, life-long treatment right from primary HIV-1 infection may not be a feasible option. In contrast, the arguments to be considered in favor of deferred treatment include improved quality of life; lower incidence of treatment-related side effects and toxicities; reduced risk of developing drug resistances; reduced overall time on medication, as well as lower overall cost of treatment [1,5]. However, a risk in deferred HAART is that the specific T helper cellular compartment may be already damaged [16]. To date, clinical studies have been unable to provide convincing data regarding the optimal timing of therapy. *In vivo *studies have often been constrained by a lack of adequate human and economic resources. Clinical trials are demanding and require long follow-up. There is also the difficulty of recruiting patients during the acute phase of the infection. In this respect, a computer model may provide a great deal of insight into the dynamics and control of HIV-infection. In particular, the use of a computer model for the simulation of HIV-infection as a predictive tool acquires a clinical and an epidemiological role, as it offers the possibility to reduce the time and the costs of antiretroviral research, potentially leading to a reduction in public health expenditure. The objective of the present analysis is to gauge by means of an immune system simulator the differences in the impact of long-term HAART when initiated either in the primary or in the chronic phase of HIV-1 infection.

There is increasing evidence that HIV-specific cytotoxic response plays a crucial role in controlling viremia [16,18-21]. CD8^+ ^T cells have been thought to mediate the decline of viremia and recent studies show that the interplay between viral replication and CD8^+ ^activity influences the equilibrium viral-set-point [16].

We found that cytotoxic responsiveness declines during long term HAART exposure in both the acute and the chronic infection stages. However, when therapy terminates, and viremia restores, *in silico *patients regain specific CD8^+ ^response. The time it takes to mount an effective natural immune response depends on the disease's progression before therapy was initiated. Computer simulations indicate that, while the immune functions are preserved during immediate therapy, the initiation of antiretroviral treatment before full emergence of a specific immune response prevents cytotoxicity from developing, whereas, in patients receiving deferred therapy, the immune system has already mounted a specific cytotoxic response in the phase prior to HAART, and is able to dump viral load and to reduce virus rebound at therapy interruption. In addition, in the presence of prompt natural immunity, the virus cannot evolve under the selective pressure of the drug (no drug escapes are detected). This difference fades within one year following discontinuation. We therefore found no valid arguments to promote very early therapy. Although other factors may explain the different immunological effects of early as compared to late initiation of HAART, our findings suggest a rationale to support deferred therapy in clinical practice. This is in line with an *in vivo *study that underlines the importance of an effective natural host-immune response [12]. It's reasonable to assume that viremia and CD8 are correlated (through a non-linear relation) to CD4^+ ^T cells since these cells constitute both a reservoir for HIV and a stimulus for CD8 cell proliferation. We suggest that a higher level of CD8 in lately treated patients with respect to those who receive early treatments is a consequence of the fact that the immune system has spent considerable time to control viremia before HAART initiates and therefore there is a higher level of cytotoxicity. However, in the simulations, the difference in viremia between immediate and deferred treated patients is very small, likewise the difference in CTLs (~10%) and therefore it is difficult to draw final conclusions. In [6] the authors find a different rebound in viremia with the early HAART controlling better the rebound.

However, there are two major differences between the cohorts used in [6] and in the present article: a) in the former, patients have been recruited with a negative western blot for anti-HIV antibodies hence before seroconversion while in the latter, cohort data includes patients classified in stage IV, V and VI with respect to [9] (see Table 2); b) in [6] patients were treated with an intensive HAART consisting of two or three NRTIs plus a NNRTI and a PI, whereas our patients were treated with just two NRTI and one PI. These two features might be the cause of a lower level of viremia at the end of HAART accounting for a slower viral rebound in [6].

Treatment interruptions are not currently recommended as routine clinical practice, although several strategies are being investigated in clinical trials. The occurrence of adverse events may, however, require cessation of therapy. At present, a large multicentric study for the treatment of primary HIV-1 infection is under investigation. This is SPARTAC (Short Pulse AntiRetroviral Therapy At HIV seroConversion), an international, randomized, controlled trial comparing three different strategies of intervention in patients recently infected with HIV-1.

The computer model used in the present analysis, the effectiveness of which has been tested in previous studies [8,22], may be considered as a predictive tool for other infectious diseases and may provide useful insights in vaccinal models that are both time consuming and costly.

## Conclusion

In conclusion, we employed a simulator of the immune system tested against clinical data aimed at identifying the golden moment to start the therapy. We observe that if the therapy starts in the acute phase (stage IV-VI of [9]) then the action of the drug impedes the immune response to develop and, as a consequence, at the end of the therapeutic period, the virus rebounds undisturbed. In contrast, if the therapy is postponed (*i.e*., beyond stage VI of [9]), then at interruption we observe a stronger immune pressure. However, in both therapeutic regime the virus completely rebounds in one year off therapy. We conclude that, given there is no therapy to date that guarantees life-long protection, deferral of therapy should be preferred in order to minimize the risk of adverse effects, the occurrence of drug resistances and the costs of treatment. Our findings could have considerable implications on the public health expenditure. Finally, it is worth to emphasize that the present analysis can hold true for patients that are diagnosticated in stage IV or beyond (*i.e*., more than about 25 days from HIV exposure). Patients treated with HAART in very early stages of the infection (stage I-III) are likely to better control viremia after treatment interruption [6]. As a future work we plan to verify if the computer simulations are in agreement with these findings.

## Methods

### Criteria for diagnosis

Twenty-two patients (21 male and 1 female) with primary HIV-1 infection were selected at the Clinical Department of the National Institute for Infectious Disease "L. Spallanzani" in Rome. Criteria for diagnosis were: documented seronegative HIV-1 antibody test within the previous 6 months; acute symptomatic seroconversion illness; evolving HIV-specific antibody response by ELISA; positive HIV-DNA PCR in PBMC, or positive plasma HIV-RNA quantification in the absence of an antibody response. Fourteen subjects were enrolled into a pilot study of Structured Therapy Interruption (STI). Two different therapy interruption protocols were used: arm A (seven patients), 4 weeks off/8 weeks on HAART; arm B (seven patients), intermittent therapy guided by plasma HIV-RNA levels, according with *pro tempore *guidelines. Specifically, patients in arm B resumed treatment only when necessary (plasma HIV-RNA > 50,000 copies/*ml*) and stopped HAART when HIV-RNA became again undetectable. This on/off treatment regime was undertaken for the first 12 months. At this time point, all patients suspended HAART and were monitored for viral and immunological parameters up to month 24. Eligibility criteria to participate in the study were as follows: CD4^+ ^T cell count ≥ 500 cells/*μl *and HIV viremia in the last two years below the detection limit, to have initiated HAART during HIV primary infection. In addition, eight patients followed unplanned treatment interruption mandating re-initiation of therapy according to medical approval (see Table 1).

The ethical committee of the institute (Clinical Department of the National Institute for Infectious Disease "L. Spallanzani" in Rome) approved this study and the patients gave a written informed consent to the blood sampling for the study.

### HIV-1 RNA determination

Plasma HIV-1 RNA levels were determined by a second-generation assay based on nucleic acid sequence based amplification (NASBA), for samples collected until 2001 and by the branched-chain DNA assay (Versant HIV RNA test, Version 3.0, lower limit of quantification 50 copies/*ml*; Bayer Diagnostics, Milan, Italy) from 2001 until 2008.

### Mathematical model

The model of immune system response we employ has been quite extensively described in [8,22-25]. In short, it belongs to a class of models that resort to bit strings to represent "binding sites" of cells and molecules, as for example lymphocyte receptors (T lymphocytes receptor TCR, B lymphocytes receptor BCR), Major Histocompatibility Complexes MHC, antigen peptides and epitopes, immunocomplexes, *etc*. [26]. The affinity among the different biological entities of the model varies according to the Hamming distance between these bit strings, *i.e*., according to their *complementarity fit*. The model includes the major classes of cells of the lymphoid lineage (T helper lymphocytes or TH, cytotoxic T lymphocytes or CTL, B lymphocytes and antibody-producer plasma cells, PLB) and some of the myeloid lineage (macrophages, MA, and dendritic cells, DC). All these entities behave as finite state machines following a set of "rules" that describe the different phases of the recognition and response of the immune system against a pathogen.

**Figure 6:**
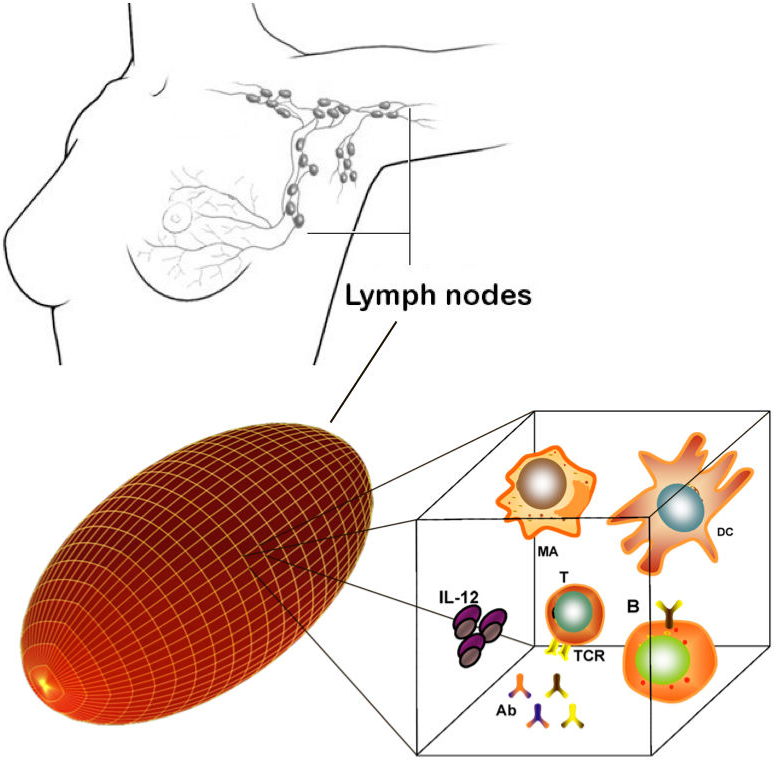
**Simulation space**. The space modeled consists in a 3D-ellipsoid lattice that resembles the typical shape of a lymph node. Each lattice point corresponds to a certain volume unit where interactions take place. Some entities are sketched: lymphocytes (T and B), antibodies (Ab), macrophages (MA), dendritic cells (DC) and the interleukins IL-12.

The model represents a single lymph node of a vertebrate animal that is mapped onto a three-dimensional ellipsoid lattice with periodic boundary conditions (Figure 6). The primary lymphoid organs thymus and bone marrow are modelled apart: the thymus is implicitly represented by the positive and negative selection of immature thymocytes before they get into the lymphatic system, whereas the bone marrow generates already mature B lymphocytes. Hence, on the lattice there are only immunocompetent lymphocytes. In order to represent the special features of the HIV infection, the model has been enhanced with the description of the *i) *HIV replication inside infected lymphocytes; *ii) *T production impairment; *iii) *specific response against HIV strains; *iv) *HIV mutation and evolution. The evolution of HIV in untreated patients has been studied and described by means of this model in [27].

The virus is represented by two bit strings (each *l *bits long); the first one corresponds to the epitope (*i.e*., the BCR's binding site) and the second one to the peptide (*i.e*., the MHC class I and II's binding site). The simulator allows the definition of an arbitrary number of epitopes and peptides. In the simulations the bit string length *l *is equal to 16 corresponding to a potential repertoire of 65536 distinct receptors and molecules. Actually, since the virus is represented by one epitope and a single peptide, each strain is identified by 2 *l *= 32 bits. So, the potential number of different virus strains becomes equal to 2^32^. Each time step of the simulation corresponds to eight hours of "real life".

While the virus and the antibodies are uniquely represented (*i.e*., they are agents like the cells), molecules with small molecular weight like interleukins or chemokines are represented in terms of concentrations and their dynamics described by the following parabolic partial differential equations plus a degradation term accounting for the finite half-life of molecules:(1)

where *c *= *c*(*x*) is the concentration of chemokines, *D *is the diffusion coefficient and *λ *is the half-life. We assume *D *= 3000 *μm*^2^/*min *and *λ *= 3 hrs [28,29]. Differences in cells mobility are taken into account as well. TH cells are the fastest ones, with an average velocity of 11 *μm*/*min*, followed by B cells with 6 *μm*/*min *and DC with a velocity of 3 *μm*/*min *[30].

#### Simulation of HAART

HAART is a *mix *of three or more powerful antiretroviral drugs, commonly reverse transcriptase inhibitors (RTIs) and protease inhibitors (PIs). Although most studies agree that HAART is not able to eradicate the virus, it is very successful in allowing the stabilization of patients' symptoms and viremia. The simulated life cycle of the virus is represented by the following stages: 1) the virus infects CD4^+ ^T cells, macrophages, dendritic cells; 2) reverse transcriptase copies the viral single stranded RNA genome into a double-stranded viral DNA. The viral DNA is then integrated into the host chromosomal DNA; 3) the virus remains at rest until an event activates the transcription; 4) the replicating virus buds from the cell membrane. Fully assembled virions are then able to infect other cells to restart the life cycle. HAART composed of transcriptase and protease inhibitors influences the life cycle of the virus as follows: RTIs block reverse transcriptase enzymatic function and avoid completion of synthesis of the double-stranded viral DNA thus preventing HIV-1 from replicating (*i.e*., it prevents the virus in stage 1 from reaching stage 2); PIs prevent viral replication by inhibiting the activity of HIV-1 protease, an enzyme used by the virus to cleave nascent proteins for final assembly of new virions (*i.e*., it prevents virus assembly in stage 4). While RTI leads to cells carrying the equivalent of the HIV-1 RNA, PI leads to "inactive" virus which is produced in infected cells and that accounts, among other things, for the HIV-1 DNA observed in real life in patients during HAART. Notice that since the clinical data available only included HIV-1 RNA counts, as an indicator of the viral rebound we use just HIV-1 RNA.

We model the damage of the CD4^+ ^T cell replenishment due to the HIV by decreasing the physiological "normal" cell count, whereas immune reconstitution due to the HAART is modeled by increasing it. The parameters of this process are set so as to reproduce, in absence of therapy, a decrease of CD4 cell count in line with clinical expectations of time to AIDS see [8] whereas immune reconstitution due to a HAART is set to achieve an average CD4^+ ^T cell increase of about 200 cells/*μl *in one year [31,32].

#### Simulation parameters setting

The parameters of the model can be classified in three categories: (1) unknown values (free parameters) which are set after a tuning procedure that starts with an initial guess and iteratively improves by looking at the outcomes that have to reproduce the well know dynamics of HIV-1 infection (*i.e*., an acute phase of primary infection lasting from three weeks to one year [33,34], an elapsed time between infection and onset of AIDS of 7 ÷ 12 years in normal progressors [33], *etc*.); (2) parameters that correspond to the initial conditions of the system and that determine the problem under investigation (for the setting of these parameters see the Section Results); (3) parameters whose value is well known and available from immunology literature. An accurate description of the all parameter setting is given as supplementary material (see additional file 1).

## Availability and requirements

An educational version of the immune system simulator is available on our website:

- Name: C-ImmSim

- Home page: http://www.iac.rm.cnr.it/~filippo/C-ImmSim.html

- Operating system(s): Linux, Unix Mac OS X, Windows

- Programming language: C

- Licence: C-ImmSim is available under a LICENSE AGREEMENT that needs to be signed: http://www.iac.rm.cnr.it/~filippo/how-to-get-cimmsim_files/LicenseAgreement-1.pdf

## List of abbreviations used

HIV: human immunodeficiency virus infection; AIDS: acquired immune deficiency syndrome; HAART: higly active antiretroviral therapy; STI: structured therapy interruption; PIs: protease inhibitors; RTIs: reverse transcriptase inhibitors; NRTIs: nucleoside reverse transcriptase inhibitors; NNRTIs: non-nucleoside reverse transcriptase inhibitors; SPARTAC: short pulse antiretroviral therapy at hiv seroconversion.

## Competing interests

The authors declare that they have no competing interests.

## Authors' contributions

FC, MB and GD contributed to the design of this study and critically revised the manuscript. PP and FC performed research, analyzed data and drafted the manuscript; RC has made sbstantial contributions to the interpretation of the results and critically revised the manuscript. All authors read and approved the final version of the manuscript including the data as presented.

## Pre-publication history

The pre-publication history for this paper can be accessed here:

http://www.biomedcentral.com/1471-2334/9/172/prepub

## Supplementary Material

Additional file 1 **Mathematical model details**. This file lists all the interactions between cells and molecules considered in the model and an accurate description of the parameter setting. Click here for file
